# Acute Kidney Injury Following Intravenous Iron Sucrose in a 67-Year-Old Male: A Case Report

**DOI:** 10.7759/cureus.88440

**Published:** 2025-07-21

**Authors:** Akhtar Purvez, Sana Khan, Emaad Abdel-Rahman, Numaan Malik, Mudhasir Bashir

**Affiliations:** 1 Pain Medicine, Lincoln Memorial University DeBusk College of Osteopathic Medicine, Charlottesville, USA; 2 Nephrology, University of Virginia, Charlottesville, USA; 3 Pulmonary and Critical Care Medicine, University of Virginia, Charlottesville, USA; 4 Psychiatry and Behavioral Sciences, University of Virginia, Charlottesville, USA

**Keywords:** acute kidney injury, chronic iron deficiency anemia, intravenous iron, iron infusion, nephrotoxicity

## Abstract

Parenteral iron therapy is a common treatment for chronic iron deficiency anemia and is generally well tolerated. We present a rare case of acute kidney injury (AKI) in a 67-year-old patient following an otherwise uneventful iron infusion. The patient was hospitalized and managed conservatively with intravenous hydration. His renal parameters returned to normal in about eight weeks. This case highlights the importance of vigilance in patients receiving parenteral iron, even in the absence of prior complications.

## Introduction

Parenteral iron therapy is frequently utilized for patients with iron deficiency anemia who do not respond to enteral iron supplementation. Modern intravenous (IV) iron formulations, such as iron sucrose and ferric carboxymaltose, have improved safety profiles compared to earlier preparations. Nonetheless, uncommon but serious adverse events, including acute kidney injury (AKI), have been documented [1,2]. IV iron works by bypassing the gastrointestinal tract and getting delivered directly into the bloodstream, where it is taken up by macrophages and used in erythropoiesis. These formulations enable the rapid replenishment of iron stores, particularly in patients with chronic illnesses or malabsorption. While generally safe, modern IV iron carries a low risk of serious adverse effects. AKI remains a rare complication, with only limited case reports describing its occurrence.

## Case presentation

A 67-year-old patient presented with complaints of abdominal pain, lower back discomfort, nausea, and marked fatigue. His current symptoms started on the night of an iron sucrose infusion for iron-deficiency anemia. They progressively worsened over three days after receiving an IV infusion of 200 mg iron sucrose (Venofer) in 100 ml of 0.9% sodium chloride over 30 minutes.

He had been diagnosed with iron-deficiency anemia a few months prior, after undergoing a thorough anemia work-up, including a complete blood count, iron studies (serum iron, ferritin, total iron-binding capacity, and transferrin saturation), and a colonoscopy to assess potential sources of blood loss. This investigation confirmed iron deficiency anemia as the underlying cause, with no evidence of gastrointestinal bleeding or other contributing pathology. He did not respond to iron supplementation, thereby supporting the decision to proceed with parenteral iron supplementation.

The patient’s past medical history was also significant for prediabetes, hypercholesterolemia, and hypertension. Medications prior to presentation include metformin, rosuvastatin, ezetimibe, lisinopril, and metoprolol. There were no recent changes in medication.

Ten days prior to symptom onset, routine laboratory tests revealed normal renal function, with a blood urea nitrogen (BUN) level of 23 mg/dL and a creatinine level of 0.7 mg/dL. Hepatic enzymes and electrolytes were in the normal range.

He was initially seen by his primary care provider, who requested repeat lab tests due to the onset of the above symptoms. Results revealed BUN elevated to 83 mg/dl and creatinine to 2.2 mg/dl, suggestive of AKI. The patient did not experience oliguria, peripheral edema, or any signs of fluid retention. Other potential causes of AKI, such as hypovolemia, nonsteroidal anti-inflammatory drug use, and contrast exposure, were clinically excluded. The patient had no recent exposure to nephrotoxins or contrast agents, and there were no signs of volume depletion.

The emergency department received his referral and admitted him to the hospital. He was managed conservatively with supportive care and IV fluids comprising 2000 mL of normal saline on the first day and 1000 mL on the second and third days. Urinalysis and imaging ruled out obstruction or infection and did not reveal proteinuria, hematuria, or casts. The fractional excretion of sodium was calculated at 1%, consistent with a prerenal etiology. Additionally, myeloma screening was conducted, including a complete blood count to assess for abnormal cells, serum free light chain assay, and lactate dehydrogenase levels, all of which were within normal limits. The patient remained hemodynamically stable throughout hospitalization.

**Figure 1:**
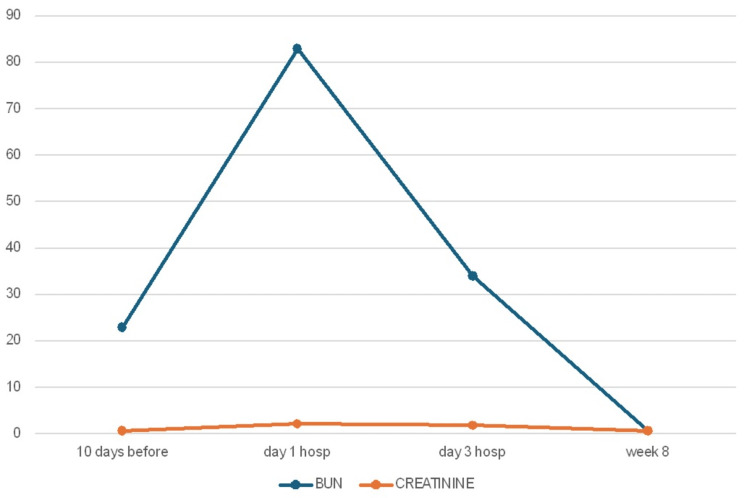
BUN and creatinine progression BUN: blood urea nitrogen

Renal parameters began to improve after two days, and he was discharged on the third day with outpatient follow-up. On the day of discharge, the lab results revealed a BUN level of 34 mg/dL and a creatinine level of 1.9 mg/dL. Repeat laboratory tests over the next six weeks demonstrated a slow but consistent decline in BUN and creatinine levels. Renal function returned to baseline after approximately eight weeks (Figure 1, Table 1).

**Table 1:** Progression of BUN and creatinine values AKI: acute kidney injury, BUN: blood urea nitrogen

Timeline/event	BUN	Creatinine	Notes
	Normal range	Normal range	
	8-26 mg/dL	0.7-1.3 mg/dL	
Baseline	23	0.7	Routine labs, normal renal function
10 days before symptoms			
Day 1 hospitalization	83	2.2	Labs suggest AKI
Day 3 discharge	34	1.9	Discharged with follow-up
Week 8	23	0.7	Renal function returned to baseline

## Discussion

Parenteral iron therapy is frequently utilized for patients with iron deficiency anemia who do not respond to oral iron supplementation. Modern IV iron formulations, such as iron sucrose and ferric carboxymaltose, have improved safety profiles compared to earlier preparations. Nonetheless, uncommon but serious adverse events, including AKI, have been documented [1,2].

In our case, the timing of symptom onset, which closely followed the iron infusion, the absence of alternative nephrotoxic exposures, and progressive improvement following supportive therapy suggest a possible causal association. The pathophysiology of IV iron-induced AKI remains incompletely understood but may involve direct tubular toxicity from labile iron, oxidative stress, or immune-mediated injury [3,4]. While a biopsy might have provided histological confirmation, it was not clinically indicated given the patient’s steady improvement without the need for invasive diagnostics.

Labile plasma iron can catalyze the formation of reactive oxygen species, which may damage renal tubular cells, particularly in patients with comorbidities such as hypertension or diabetes that predispose the kidneys to microvascular injury [5]. Statins, angiotensin-converting enzyme inhibitors, and other antihypertensive medications have not been implicated in such episodes, although underlying chronic endothelial stress may increase susceptibility to injury.

Our findings complement the existing literature that has raised concerns about the nephrotoxic potential of IV iron. The capacity of IV iron, particularly nondextran formulations like iron sucrose, to generate oxidative stress and renal injury independent of inflammatory markers has been demonstrated. These observations suggest that renal damage may occur via dual pathways: oxidative and direct tubular toxicity. They noted that proteinuria was more commonly observed with iron sucrose, lending plausibility to its role in renal injury [6].

IV iron administration can lead to renal structural and functional injury, with iron sucrose implicated in increased proteinuria and impaired renal outcomes [7]. This highlights the potential for short-term renal insult from iron infusions, especially in patients with predisposing factors such as endothelial dysfunction.

Compared to these studies, our case contributes unique clinical value by documenting a clear temporal trajectory, from infusion to symptom onset, AKI confirmation, and complete recovery, along with comprehensive exclusion of other etiologies. Notably, there was no proteinuria. While not definitive, this case adds to the body of evidence suggesting that even a single dose of iron sucrose can precipitate AKI in susceptible individuals. Heightened vigilance, close monitoring of renal function, and awareness of potential post-infusion symptoms are warranted.

While rare, the risk of AKI post-iron infusion warrants heightened clinical vigilance, particularly in patients with previous cumulative exposure, advanced age, or underlying renal vulnerability. Monitoring renal function before and after infusion in high-risk individuals may allow for early detection and intervention.

## Conclusions

This case illustrates the value of considering rare but significant adverse effects of iron infusions, particularly AKI. Vigilant monitoring of renal function before and after parenteral iron therapy may be warranted in high-risk patients or those who develop new symptoms following infusion. Given the increasing use of IV iron in outpatient settings, clinicians should maintain a high index of suspicion for renal complications, especially in older adults with underlying comorbidities such as hypertension or diabetes.
